# Pea and Lentil Flours Increase Postprandial Glycemic Response in Adults with Type 2 Diabetes and Metabolic Syndrome

**DOI:** 10.3390/foods14111933

**Published:** 2025-05-29

**Authors:** Donna M. Winham, Mariel Camacho-Arriola, Abigail A. Glick, Clifford A. Hall, Mack C. Shelley

**Affiliations:** 1 Department of Food Science & Human Nutrition, Iowa State University, Ames, IA 50011, USA; marielcamarr@gmail.com (M.C.-A.); aglick03@gmail.com (A.A.G.); 2 Department of Dairy and Food Science, South Dakota State University, Brookings, SD 57007, USA; clifford.hall@sdstate.edu; 3 Departments of Political Science and Statistics, Iowa State University, Ames, IA 50011, USA; mshelley@iastate.edu

**Keywords:** legumes, pulses, plant-based foods, glucose, gluten free, satiety, flatulence, flour, carbohydrates, functional ingredients

## Abstract

Pea and lentil flours are added to baked foods, pastas, and snacks to improve nutritional quality and functionality compared to products made solely with refined wheat flour. However, the effect of whole pulses versus their serving size equivalent of flour on blood glucose has not been investigated in persons with altered glycemic response. Health claims for whole pulses are based on a ½ cup amount whereas commercial pulse flour servings are typically a smaller size. The glycemic responses of four treatment meals containing 50 g available carbohydrate as ½ cup whole pulse or the dry weight equivalent of pulse flour were compared with a control beverage (Glucola^®^). Eleven adults with type 2 diabetes mellitus (T2DM) and eight adults with metabolic syndrome (MetS) completed the study. Venous blood samples were collected at fasting and at 30 min intervals postprandial for three hours. Changes in net difference in plasma glucose over time from baseline and incremental area under the curve (iAUC) segments were analyzed. All four pulse meals attenuated the iAUC compared to the control from 0 to 120 min for T2DM participants and 0–180 min for MetS participants. Whole pulses produced a lower glycemic response than pulse flours in the early postprandial period for persons with T2DM and during the overall test period for those with MetS.

## 1. Introduction

Type 2 diabetes mellitus (T2DM) is a condition characterized by high blood glucose levels due to inadequate or ineffective utilization of insulin by the body [1]. Elevated blood glucose can damage the vascular system, kidneys, eyes, and other organs if not controlled over time. Risk factors include being overweight, sedentary lifestyle, older age, and experiencing under or over nutrition in utero [2]. For 2021, the global prevalence for T2DM was estimated at 529 million people [3]. Low-income and middle-income countries have higher percentages of people with T2DM. The global nutrition transition has contributed to higher dietary intakes of added sugars, refined carbohydrates, and hyper palatable foods [4]. Other drivers, such as the social determinants of health, limited access to healthcare, socioeconomic status, and gene–environment interactions contribute to the development of T2DM [2]. As of 2019, it is estimated that T2DM affects 37.3 million adults (11.3% of the US population) [1], leading to a healthcare expenditure of 415 billion USD in the United States. Metabolic syndrome (MetS) is defined by the presence of at least three out of the following five conditions: high blood glucose, hypertension, high triglycerides, low high-density lipoprotein (HDL) cholesterol, and large waist circumference [5]. Individuals with MetS are at higher risk of developing T2DM [5].

With the above risks related to glycemic response, there is interest in replacing or supplementing refined carbohydrates in the diet with pulses, which are known to enhance blood glucose regulation and satiety [6]. Pulses are diverse dry seed crops such as lentils, dry peas, dry beans, chickpeas, and others. They are high in protein and dietary fiber and are rich sources of shortfall nutrients such as folate, iron, zinc, and potassium [7,8]. Glycemic response benefits have consistently been observed with pulse consumption when they are provided as whole (intact) pulses alone or as part of a meal [9]. A reduced glycemic response following pulse consumption is due in part to the sturdy cotyledon cell wall, which stays unbroken through cooking and limits access to starch granules [10,11]. Whole pulses, alone or as part of a meal, lower postprandial glycemic response in normoglycemic adults and persons with T2DM and MetS [12,13]. Increased satiety after whole pulse consumption has been reported [6]. Furthermore, a positive effect on weight management has been reported in individuals with T2DM and MetS [14].

The interest in incorporating pulses in the diet expands beyond their effect on glycemia. Pulses are naturally gluten-free and are suitable for plant-based diets. They have a protein digestibility score similar to that of meat [15]. When pulses are consumed on the same day with cereal grains, their amino acid profiles complement each other resulting in complete proteins [16]. Beyond nutritional quality, pulses have a positive environmental effect and lower production costs for farmers since they require less fertilizer, less water, and improve soil for the next crop rotation [17]. Subsequently, these characteristics lower costs for food product development [17]. Lentil flours can reduce greenhouse gas emissions by 17% when integrated into breakfast cereal products. In turn, pea flour is calculated to reduce emissions by 12% when implemented into these products [18].

Given these advantages, the incorporation of pulses into products traditionally composed solely of cereal grains, such as baked foods, is a promising opportunity for food companies and consumers alike [19]. Demand for pulse flour as an ingredient in processed food has risen in the US and internationally [20]. By 2033, the US pulse flour market is expected to grow by 5.06% compared to the anticipated 3.4% for the wheat flour market [21,22]. As a relatively new industry, pulse milling is in the process of developing target protein concentrations and precise specifications for particle size [23]. Innovations in milling are being explored to modify the fiber and protein levels, obtain the best particle sizes for functionality, and reduce starch damage and off-flavors in pulse flour [24,25]. Different milling techniques will produce flours with variable particle sizes, which can alter the glycemic response of the food products [25].

However, compared to whole pulses with an intact cell wall, pulse flour can cause an increased glycemic response since the cell wall is broken down [26]. When raw beans are milled into flour, their cellular components are more accessible to digestive enzymes and contain more free starch, which results in an elevated blood glucose response compared to whole-boiled beans in one study [26]. Given that many consumers know the health and nutritional benefits of whole pulses, it is essential to understand whether the same attributes apply to pulse flours too [27].

A handful of studies have evaluated the glycemic response of pulse flour in healthy adults, with variable findings. Milled pulses appear to elevate postprandial glycemic response in normoglycemic individuals as part of a meal [28]. Similar findings are observed when milled pulses are made into pasta products [29], pulse powders [30], or pulse fractions [31] in normoglycemic people as compared to whole pulses. While pulse flour may be less effective than whole seed, they still do reduce the glycemic response when compared to original wheat or oat products [31,32].

Chickpea flour added to wheat bread produced a significantly lower glycemic response compared to wheat alone [33], with a trending reduction in glucose area under the curve [34] for two separate acute trials. Chamoun et al. found that replacing 25% of wheat flour content in muffins with pureed red or green lentils significantly reduced glycemic response in normoglycemic participants [35]. Conversely, Fujiwara et al. found that wheat pasta with up to 50% pea and lentil flour produced a lower glycemic index compared to 100% wheat flour pasta, yet this substitution had no impact on glycemic response [36]. Green lentil-derived flour (roasted and spray dried) elicited the highest glucose response of the lentil-based treatments as compared to a potato flake control [37]. The lower dietary fiber content and smaller particle size of the lentil flour-based treatment may have been responsible for the lack of glycemic control.

Factors affecting glycemic response results in various studies include the pulse type and the serving size of whole pulses versus milled pulse flour. The majority of health benefits for management of blood glucose, cardiovascular risk, and satiety are based on a ½ cup or 100 g serving size of whole pulses [38]. However, most servings sizes of commercial pulse flour foods contain much less than a ½ cup serving.

While whole pulses increase feelings of satiety, it is unclear whether products made with pulse flours have the same effect. Research suggests that lentil pastas resulted in higher satiety compared to durum wheat pasta, yet no comparisons to whole lentils were made [6,39]. No differences in appetite were found between boiled whole black beans and three 100% black bean pastas among normoglycemic participants [29].

There may be barriers to consumption of pulses among individuals concerned with gastrointestinal discomfort and flatulence. The high fiber content of whole pulses may be responsible for increased flatulence, bloating, and stool changes in persons with low dietary fiber intake [40]. When examining motivators or barriers to whole bean consumption, Doma et al. noted flatulence and abdominal discomfort were the second most reported barrier [41]. However, this perception may be over-emphasized because of social sensitivity. Symptoms are seemingly transient after more frequent consumption of whole pulses [40]. Little is known about consumer experiences or their perceptions of gastrointestinal symptoms associated with pulse flour products. No significant increases in flatulence, bloating, or stool changes were observed in a clinical trial with 100% black bean pastas containing 19–22 g of fiber per test meal [29]. The test meal alone was close to the daily fiber recommendation for most people [42].

Previous studies have focused primarily on normoglycemic consumers. Therefore, the effect of pulse flours on glycemia in individuals with altered or impaired glycemic metabolism remains largely unknown. Individuals with T2DM and MetS may seek out processed foods containing pulse flour thinking these will be metabolized like whole pulses. Increased knowledge of how pulse flour influences glycemic response is necessary to reduce potential health risks. To address these questions, in part, the current study objectives were to compare the glycemic and satiety responses of ½ cup of whole lentils, whole dry peas, and their flour equivalents to a glucose control beverage (Glucola^®^) in adults with T2DM and MetS. The following hypotheses were investigated: (1) all four pulse meals would result in a lower postprandial glycemic response than the control beverage, and (2) whole pulses would lower the glycemic response more than their pulse flours. An exploratory objective was to evaluate the magnitude of differences in glycemic response between individuals with T2DM and MetS. The study was approved by the Iowa State University Institutional Review Board (#17-191) and registered at ClinicalTrials.gov (#NCT05145998).

## 2. Materials and Methods

### 2.1. Study Design

The unblinded, semi-randomized crossover study included five treatments: (1) Glucola control beverage; (2) whole lentils; (3) lentil flour; (4) whole peas; and (5) pea flour. Participants with T2DM or MetS were first randomized to begin with lentils or peas, and next randomized within this pulse type to either the whole or flour form first for treatment order. A priori power analysis for this 5 × 5 crossover study supported a sample size of 10 participants; this was satisfactory for a medium effect size (d = 0.50) at 80% power, with a Type I error level of 0.05 for this seven-timepoint trial.

### 2.2. Study Recruitment and Selection Criteria

Recruitment was conducted through posted flyers, newspaper ads, Facebook community posts, and e-mail or listserv announcements. Interested people completed an online form or oral interview to assess pre-eligibility. The selection criterion for the T2DM group was a physician diagnosis at least 4 months prior to their study start date. MetS participants were identified during screening as those with elevated glucose (≥5.6 mmol/L, HbA1c 5.7–6.4%) and two or more of the following National Cholesterol Education Program Adult Treatment Panel III criteria: elevated waist circumference (>102 cm for men, >89 cm for women), blood pressure >130/85 mmHg, fasting triglyceride concentrations >1.7 mmol/L, and low fasting HDL concentrations (<1.0 mmol/L for men, <1.2 mmol/L for women) [43]. Participants with T2DM were eligible if they managed their condition with metformin, Trulicity^®^ (a once-a-week injectable GLP-1 receptor agonist), or diet and exercise. These medications were allowed due to the long-term background effect of metformin on blood glucose, and the short-term, yet consistent, effect of Trulicity to control glucose consistency over time [44]. Other inclusion criteria included an age between 24 and 75 years, body mass index (BMI) 22–39.9 kg/m^2^, HbA1c % ≤ 10% for the T2DM cohort and <6.4% for the MetS group, being ambulatory, and the ability to conduct activities of daily living independently [45]. Exclusion criteria included tobacco or nicotine delivery products, food allergies or intolerances, pregnancy, lactation, uncontrolled hypertension, diagnosed gastrointestinal (GI) disease, consumption of a salt- or sodium-restricted diet, and 10% or more weight fluctuation over the previous 6 months. Medications that do not have known influences on glucose or insulin metabolism were permitted. Current dosages needed to be stable for ≥6 months. Medication dosage changes were not allowed during the study. Additional study details and protocols can be found in Camacho-Arriola, 2020 [45].

Seventeen T2DM and 12 MetS people were invited to an in-person meeting to learn about the research protocols and expectations. During the information session, a team of researchers demonstrated how to complete the pre-test-day 24 h food logs, appetite measures survey, and gastrointestinal symptom questionnaire. For the pre-test day evening meal, potential participants had three commercial frozen dinners to choose from, and two optional pre-packaged cookie selections. Participants were instructed to eat the study-provided pre-test evening meal 12 h before their morning test time. The same meals were consumed for all five pre-test evenings.

After signing the consent form, participants were taken to an examination room for blood pressure and anthropometric measurements taken in light clothing and without shoes. Blood pressure was measured using an Omron automatic digital blood pressure monitor (Omron Healthcare, Inc., Lake Forest, IL, USA) after participants sat quietly for 5 min. A wall-mounted stadiometer (Model 216, Seca, Chino, CA, USA) was used to determine height to the nearest 0.1 cm. Waist circumference was assessed with a nonelastic measuring tape to the nearest 0.1 cm (Seca 201, Seca, Chino, CA, USA). Using a digital scale, body weight was recorded to the nearest 0.1 kg (Detecto, Webb City, MO, USA). A fasting venous blood sample was drawn from the antecubital region of the forearm by a registered nurse for analysis of hemoglobin A1c (HbA1c) for both the T2DM and MetS study candidates. The potential MetS group had measures of triglycerides, low-density lipoprotein (LDL), high-density lipoprotein (HDL), and complete blood count (CBC) at screening to assess the presence of MetS characteristics [43]. A commercial laboratory analyzed samples for HbA1c via an immunoturbidimetry assay. Lipid panel items and the CBC were determined with an automated hematology analyzer (Quest Diagnostics, Wood Dale, IL, USA).

For the seventeen T2DM people who were screened, one was not diabetic, two declined to participate further, and three were excluded for medical reasons (medication change, anemia, scarred veins). Of the twelve MetS potential participants screened, one declined to participate further, one was normoglycemic, one had T2DM, and one had difficulty with blood draws. Eleven people with T2DM and eight with MetS successfully completed the five test days.

### 2.3. Lentil and Pea Cultivars and Their Flour Production

Commercially available lentils and peas may be a mixture of seeds from different market classes and geographic areas. There are differences in nutrient composition even within the same market class based on variety and growing environment [46]. For these reasons, it is best practice for clinical trials to be conducted with known varieties that are grown in the same environments to reduce confounding effects on the nutrient composition of the seeds. Avondale green lentils (*Lens culinaris*; Plant Variety Protection Office (PVP) number 201400093, issued 31 March 2015) and Hampton dry green peas (*Pisum sativum*; PVP 201500303, issued 6 July 2016), were sourced by the USA Dry Pea & Lentil Council for the study. Both cultivars have high yield potential compared to other commercial varieties and were grown on the same Montana farm [47,48].

Whole lentil and peas were processed into flours at North Dakota State University (NDSU). To maximize similarity of the flours to the whole pulse for the study purpose, seeds were not dehulled before processing in contrast to common commercial practices for pulse flours [49]. First, pulses were soaked overnight at 25 °C in water (10 parts water to 1 part pulse). Soaking was performed to soften the pulse hull and hydrate the cotyledon [23]. Second, pulses were drained over a 40-mesh sieve (Gilson Inc., Lewis, OH, USA). Material that passed through the screen was discarded. Next, pulses were distributed in single layers on perforated baking pans in single layers (approximately 0.45 kg per tray). A Baxter OV300G Mini Rotating Rack Convection Oven (Baxter Manufacturing Co., Orting, WA, USA) set at 149 °C for 18 min (lentil) or 33 min (peas) to complete the heat treatment. The pulses were mixed at 5 min intervals until the end of the heating time.

After the mixing step, the heat-treated pulses were milled in a two-step system with a roller mill (roll stands by Creason, Wichita, KS, USA; rolls by Buhler AG, Uzwil, Switzerland). The first roller pass dehulled the pulses, and the second pass reduced the size of the cotyledon. The first pass was at 0.7 kg/min feed rate with corrugated rolls (8% spiral, 0.1 mm land, 8.9 flutes per cm, 0.254 mm roll gap) using sharp-to-sharp action and a front/back roll speed differential of 1:2.5. This process created hull and cotyledon fractionation. The second pass, which was at 0.3 kg/min feed rate with smooth rolls (0.038 mm roll gap) and a 1:1.23 front/back roll speed differential, was performed on the cotyledon fraction. Hulls obtained from the first pass in the roller mill or the break roll were subsequently passed through a hammer mill (Model DASO6, Fitzpatrick, Elmhurst, IL, USA) at 102 m/s hammer speed, and 0.838 mm diameter screen aperture.

The hulls were fed back into the pulse samples after milling, and the mixture sifted through 80-mesh and 100-mesh sieves. The particle size distribution for the lentil and pea flours is shown in Table 1. An 80-mesh sieve was used for above 177 µm, and a 100-mesh sieve was used for the other particle size ranges. Particle size classifications are highly variable in the literature [23,24]. The flours used in this study had fine to intermediate particles, depending on the classification used.

### 2.4. Whole Pulse vs. Pulse Flour Equivalency Calculations

Whole pulse and pulse flour portions were calculated to be equivalent to ½ cup dry weight serving of pulses [42]. The computations were made based on the percentage of moisture compared to dry solids per 100 g. Values used were from the proximate analysis provided by Eurofins Scientific Incorporated (Des Moines, IA, USA), and all weights were in grams. A ½ cup serving of each whole pulse treatment was determined. The dry solid weight per 100 g of each whole pulse and pulse flour was calculated using Equation (1), and the amount of dry whole pulse solids per 1 cup was calculated using Equation (2). The amount of flour required for an equivalent ½ cup dry weight serving to that of the whole pulse counterpart was derived from Equation (3). Calculations with the actual test values are shown in Supplemental Table S1. For cooked Avondale lentils, the whole ½ cup weight was 80.0 g, and the flour equivalent was 40.1 g. The cooked Hampton pea ½ cup serving was 76.0 g, and 32.8 g was the equivalent for the flour.

(1) $$Dry\;solid\;weight\;\left(g\right)=100\;(g)-sample\;moisture\;(g)$$

(2) $$\frac{dry\;whole\;pulse\;solids\;(g)}{1\;cup}=\frac{grams\;of\;cooked\;whole\;pulse}{1\;cup}\times \frac{dry\;solid\;weight\;(g)}{100\;g}$$

(3) $$\frac{Flour\;(g)}{1\;cup}=\frac{dry\;pulse\;solids\;per\;1\;cup}{dry\;solid\;weight\;of\;flour}$$

### 2.5. Nutrient Analysis of Pulses and Test Meal Components

Eurofins Scientific Incorporated Nutrition Analysis Center (Des Moines, IA, USA) conducted the proximate analysis of cooked pulses and flours (whole lentil, lentil flour, whole pea, pea flour) and test meal components (spaghetti sauce, and wheat bread). Two samples of each food item were analyzed, and the average was reported. Per Eurofins, the standard Association of Official Analytical Chemists (AOAC) methods were used for total fat (AOAC 954.02) [50], crude protein/total nitrogen (AOAC 992.15; AOAC 990.03) [51], moisture (AOAC 925.09) [52], ash (AOAC 942.05) [53], total starch (AOAC 996.11) [54], total dietary fiber (TDF; AOAC 991.43) [55], calories (CFR—Atwater calculation) [56] and total carbohydrates (CFR 21-calculation) [56]. Available carbohydrate was derived from the calculation: total carbohydrates—TDF.

The four treatment meals contained approximately 50 g of available carbohydrates (Table 2). The gram weights for each of the pulses and meal components are shown in Table 2. Pulse treatments were mixed with 167 g of Classico Traditional spaghetti sauce (The Kraft Heinz Company, Glenview, IL, USA). Spaghetti sauce served as a simple food matrix for the pulses. The two flour meals had 83 g (~1/3 cup of water) mixed in last for palatability, as the flours tended to thicken the sauce. The T2DM cohort was served 49 g or about 1 slice of Honey Whole Wheat Grain bread (Pepperidge Farm, Norwalk, CT, USA). The whole pea and pea flour meals for the T2DM participants had 4 g of sugar added to meet ~50 g available CHO content. Midway through the T2DM study, a miscalculation was found in the pea test meal nutrient composition spreadsheet. The 4 g of sugar was unnecessary to meet the 50 g available CHO. Since the T2DM study was underway, meal composition was maintained. For the MetS cohort, the bread amount was modified to 45 g, and no sugar was added to the pea treatments. This change lowered the available CHO slightly for the MetS cohort meals. The differences in available CHO between treatments and between cohorts were not statistically significant.

### 2.6. Subjective Appetite and Gastrointestinal (GI) Symptoms Survey

For each pre-test day, participants completed subjective appetite surveys before and after the midday meal. Pre-test day data collection familiarized participants with their sensations of fullness and details of the form. A 100 mm visual analog scale was used to quantify participant sensations at baseline and at 60-, 120-, and 180 min following test meal consumption. The scale range was anchored with opposing statements of “not at all” to “as much as I ever felt” [57]. Questions included hunger, fullness, satiation, desire to eat, and the volume that one perceived that they could eat [57]. The subjective appetite score was computed as: hunger + desire to eat + volume one could eat + (100 − fullness score) divided by 4 [6].

GI symptom surveys were completed on the pre-test day evening and the evening of the test day between 18:00 and 21:00. Survey questions included changes in flatulence, bloating, and whether symptoms interfered with normal daily activities [29,40]. Changes in GI symptoms were described as increased or decreased using a scale from 0 to 5, representing “little change” to “a lot of change”, or “a little more frequent” to “much more frequent”.

### 2.7. Participant Test Day Procedures

In the 24 h before testing, participants completed an all-day food log, appetite measures for their midday meal, and a gastrointestinal questionnaire in the evening. Participants were to avoid moderate or vigorous exercise, caffeine, alcohol, and eating other pulses. They consumed the provided evening meal 12 h before testing. The nutrient analysis of the participants’ pre-test daily meals is shown in Supplemental Table S2.

Upon participant arrival, a staff member reviewed pre-test day forms and confirmed protocol compliance regarding fasting, dietary intakes, and exercise limits. Food log and 24 h recalls, anthropometric measurements, blood draws, and study compliance are all previously described [45]. Fasting venous blood samples were collected for blood glucose levels at time 0, and once the meal was consumed, further measurements were taken at 30, 60, 90, 120, and 180 min post-treatment. Participants were required to consume the test meal within 12 min while being observed. Bottled water was provided ad libitum, and the amount consumed over the 3 h test period was recorded.

### 2.8. Test Meal Preparation

The test meal ingredients were weighed and cooked each morning at the testing facility. The 50 g Glucola control beverage was stored in a walk-in cooler at 4 °C. After being inverted to mix contents, the drink was served to the participant in a clear glass. The whole peas and whole lentils were soaked (~16 h and ~2 h, respectively) at room temperature (20 °C) in reverse osmosis (RO) water in a 1:3 ratio. The whole pulse was added to boiling RO water (100 °C). Whole lentils simmered for approximately 12 min, whereas the whole peas were cooked for approximately 1 h. Both of the whole pulses were sampled for tenderness over the cooking process. The ideal consistency was slightly soft, but still firm.

The test pulses were mixed in pasta sauce and served with toasted bread on the side. A whole slice of bread was weighed to the study weight (49 g T2DM; 45 g MetS; Table 2), and toasted in a Proctor Silex toaster (Hamilton Beach Brands, Glen Allen, VA, USA) at the medium setting for approximately two minutes. In a separate bowl, 167 g of spaghetti sauce was heated in a 1300 W Panasonic microwave (Panasonic, Kadoma, Osaka, Japan) at 100% power for 15 s, with a lid to prevent moisture loss. The sauce was stirred, and heating was repeated two additional times for a total of 3, 15 s microwave treatments. Freshly cooked pulses were weighed out separately, then added to the heated sauce. For pulse flour treatments, 83.3 g of RO water was heated in the microwave for 1 min. The pulse flour was weighed and mixed with the RO water and spaghetti sauce. Meals were served promptly to participants.

### 2.9. Data Transformations and Statistical Analyses

The five pre-test 24 h food logs were entered into and analyzed for macro and micronutrient intakes by Food Processor (version 11.3, ESHA Research, Salem, OR, USA). Data were evaluated for distribution normality using the Shapiro–Wilk test, descriptive statistics, and visual histogram inspection. No data were transformed. Mean imputation was used for four missing plasma glucose net-change concentrations out of 95 total (4/95; 4.2% of cases) [58]. Repeated measures general linear models were estimated to assess glucose and subjective appetite score mean differences between treatments over time. Greenhouse-Geisser corrections of within-subject effects *p*-values were used in the case of unequal variances (Mauchly’s test *p* < 0.05). A one-way analysis of variance (ANOVA) was used to compare means for glucose net change at timepoints, incremental area under the curve (iAUC) for each treatment, and sensory measures. Tukey HSD post hoc testing was then applied to pinpoint differences between specific treatments. Timepoint differences between fasting and post-treatment glucose concentrations (0–60 min, 0–120 min, and 0–180 min) were determined and iAUC calculations were completed using the trapezoidal rule [59]. IBM SPSS version 26 (IBM, Armonk, NY, USA) was used for all statistical analyses.

## 3. Results

### 3.1. Participant Characteristics

Descriptive statistics at study entry for the 19 participants (9 women, 10 men) are shown in Table 3. All participants self-identified as White. The mean age was 51 years. T2DM participants tended to be older (mean age of 55 years) than participants with MetS (44 years). The majority of T2DM participants (*n* = 9) used metformin to manage their diabetes, with one using Trulicity, a GLP inhibitor, and one using dietary methods and physical activity. There were no significant differences in body weight, BMI, and blood pressure measurements between test days for all participants, nor between T2DM and MetS subgroups overall. In Table 3, the variation around the mean as the standard error of the mean (SEM) is indicated, which measures the precision of the estimate of the mean by taking into consideration the sample size (*n* = 19), in contrast to the standard deviation which does not adjust for the amount of information on which the estimated mean is based. A smaller SEM indicates a more precise, and hence more meaningful, estimate of the mean.

Of the 8 MetS participants, 1 met three criteria, 4 met four criteria, and 3 met all five NCEP ATP III criteria for metabolic syndrome. Mean BMI (30.0–34.9) did not differ between groups, with all participants in the overweight range or greater [43]. According to NIH guidelines, the majority of participants had high triglyceride concentrations and waist circumference measures [60]. Participants with T2DM had greater plasma glucose concentrations at screening (*p* = 0.046), with HbA1c trending toward significance compared to those with MetS (*p* = 0.07). Total and LDL cholesterol (LDL-C) were significantly greater among those with MetS (both *p* = 0.01).

### 3.2. Postprandial Glucose Response

#### 3.2.1. Glucose Differences over 30-Min Increments

**Figure 1:**
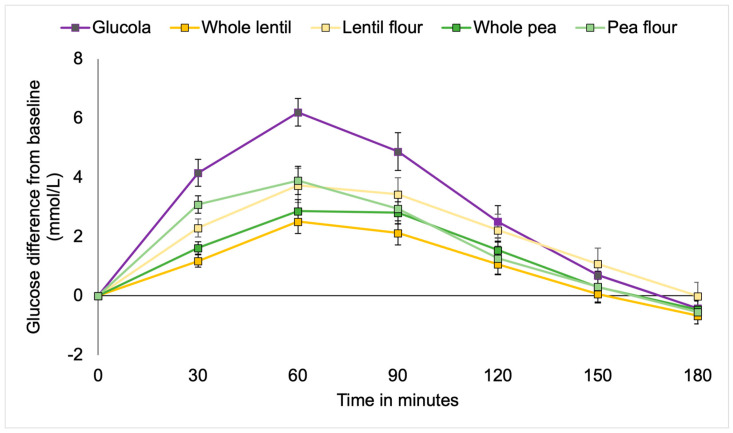
Incremental changes in plasma glucose among participants with type 2 diabetes.

Plasma glucose concentrations (mean ± SEM) are presented as net change from fasting values (Table 4). No significant differences in fasting blood glucose were observed for individual participants over the five test days. For the T2DM cohort, plasma glucose concentrations were significantly different at the timepoints over the 180 min testing period by study treatment [F(2.267, 113.344) = 194.418, *p* < 0.001; partial eta^2^ = 0.795] (Table 4, Figure 1). A significant interaction between time and meal types was observed [F(9.067, 113.344) = 5.382, *p* < 0.001; partial eta^2^ = 0.301]. Between-subject effects [F(4, 421.565) = 4.316, *p* = 0.004; partial eta^2^ = 0.257] explained about 25% of the observed variance. Multiple comparison analysis supported significant differences between the Glucola control and whole lentil (*p* = 0.003, 95% confidence interval (95% CI 0.53 to 3.39) and whole pea (*p* = 0.027; 95% CI 0.13 to 2.99). No significant differences were found between the pulses by type or form overall.

Significantly higher mean blood glucose levels occurred with the control treatment at 30 min as compared to whole lentils and whole peas (both *p* < 0.001) and lentil flour (*p* = 0.001), but not pea flour. The pea flour elicited significantly higher blood glucose than the whole lentils (*p* = 0.001) and whole peas (*p* = 0.012). At the 60 min timepoint, the average glucose was significantly lower with whole lentils and whole peas (both *p* < 0.001), lentil flour (*p* = 0.004), and pea flour (*p* = 0.009) than the control. There were no differences between the pulse treatments for the remainder of the timepoints. At 90 min postprandial, blood glucose levels in participants consuming whole lentils (*p* = 0.003) and whole peas (*p* = 0.043) were lower than the Glucola control, with pea flour trending lower at *p* = 0.069. No differences were observed between the control and treatments during the remainder of the test period for the T2DM cohort (Table 4, Figure 1).

**Figure 2:**
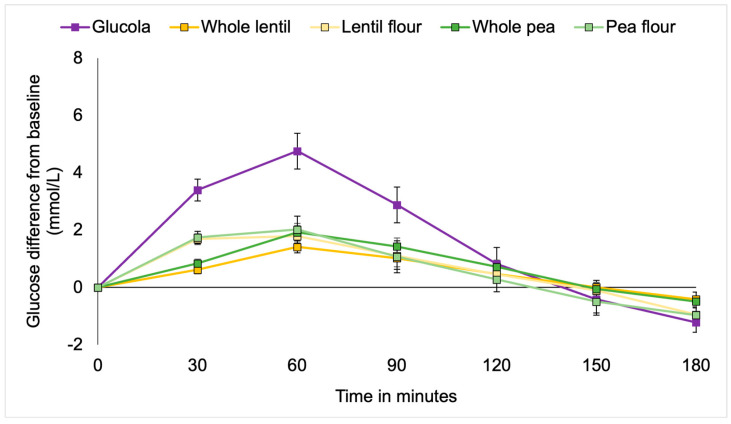
Incremental changes in plasma glucose among metabolic syndrome participants.

MetS participants showed a similar pattern to the T2DM cohort. Plasma glucose concentrations for the MetS participants were significantly different at the timepoints over the 180 min testing period by study treatment [F(2.146, 113.891) = 90.939, *p* < 0.001; partial eta^2^ = 0.722] (Figure 2). A statistically significant interaction between time and meal types was observed [F(9.087, 113.891) = 5.992, *p* ≤ 0.001; partial eta^2^ = 0.406]. Between subject effects [F(4107.285) = 3.701, *p* = 0.013; partial eta^2^ = 0.297] explained almost 30% of the observed variance.

Multiple comparison analysis detected statistically significant differences between the Glucola control and each of whole lentils (*p* = 0.017; 95% CI 0.16 to 2.21), pea flour (*p* = 0.032; 95% CI 0.07 to 2.12), and lentil flour (*p* = 0.046; 95% CI 0.1 to 2.07). There were no significant differences between the pulses by type or form overall. Post hoc analysis by timepoint showed significantly higher net glucose at 30 min with the Glucola control treatment compared to all lentil and pea meals (*p* < 0.001). Whole lentils were significantly lower than pea flour (*p* = 0.012) and lentil flour *p* = 0.019). There was evidence of a trend toward a statistically significant difference between whole peas and pea flour (*p* = 0.065). At the 60 min duration, blood glucose levels in participants consuming the control treatment remained significantly higher than after consuming the whole lentils, lentil flour, whole peas (all *p* < 0.001), and pea flour (*p* = 0.001) treatments. The four treatments were not significantly different from each other for the remainder of the timepoints. Whole lentils (*p* = 0.057), pea flour (*p* = 0.068), and lentil flour (*p* = 0.076) trended lower than the control treatment at the 90 min time but not at later measurement intervals (Table 4, Figure 2).

#### 3.2.2. Incremental Area Under the Curve (iAUC)

**Figure 3:**
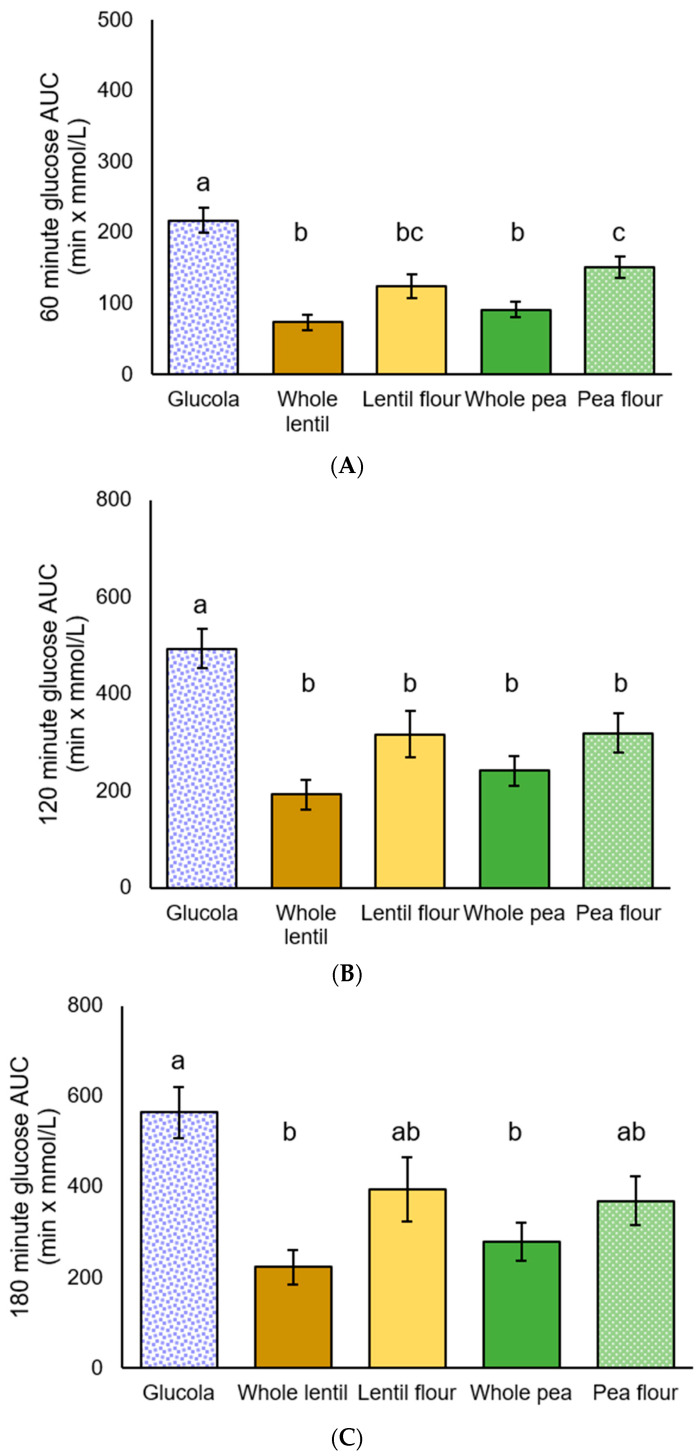
Plasma glucose incremental area under the curve (iAUC) for participants with T2DM: (**A**) 60 min, (**B**) 120 min, and (**C**) 180 min after the test meal consumption; data are presented as mean ± standard error of the mean. Letters that differ (a–c) indicate significant differences (*p* < 0.05) between treatments.

Significant differences in iAUC glucose response were observed at all durations by ANOVA [F(4,50) = 15.304, *p* < 0.001], [F(4,50) = 8.922, *p* < 0.001], [F(4,50) = 5.923, *p* = 0.001]. Post hoc analysis showed the control beverage produced a significantly greater glucose response among T2DM participants from 0 to 60 min than for whole lentils, lentil flour, whole peas (all *p* < 0.001), and pea flour (*p* = 0.017). The 0 to 60 min iAUC results for whole lentils (*p* = 0.004) and whole peas (*p* = 0.042) were significantly lower than for pea flour. For the 0 to 120 min duration, the control beverage iAUC was greater than whole lentils and whole peas (both *p* < 0.001), lentil flour (*p* = 0.016), and pea flour (*p* = 0.018). The control beverage iAUC from 0 to 180 min was significantly higher than for whole lentils (*p* < 0.001) and whole peas (*p* = 0.004). No significant differences were observed between the pulse treatments for the 120- and 180 min increments (Table 5, Figure 3).

**Figure 4:**
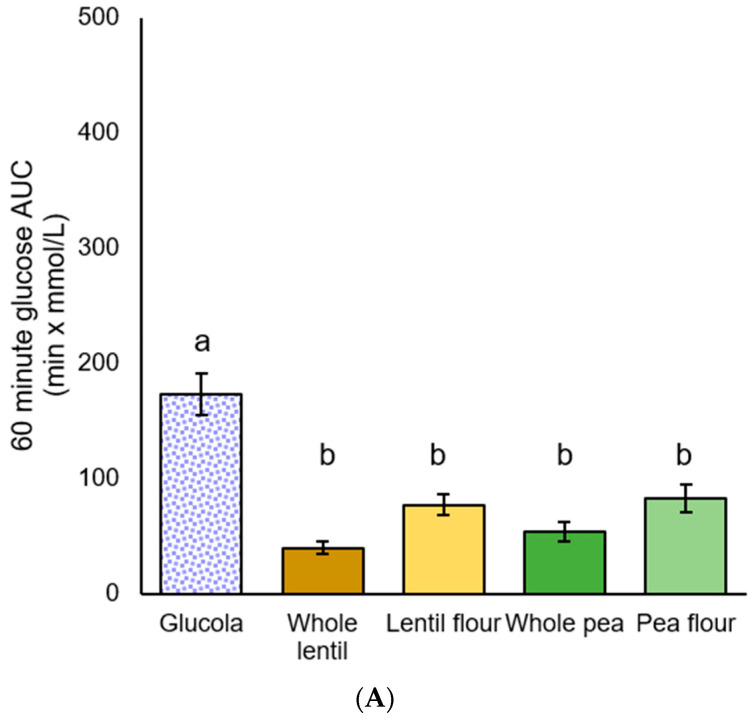

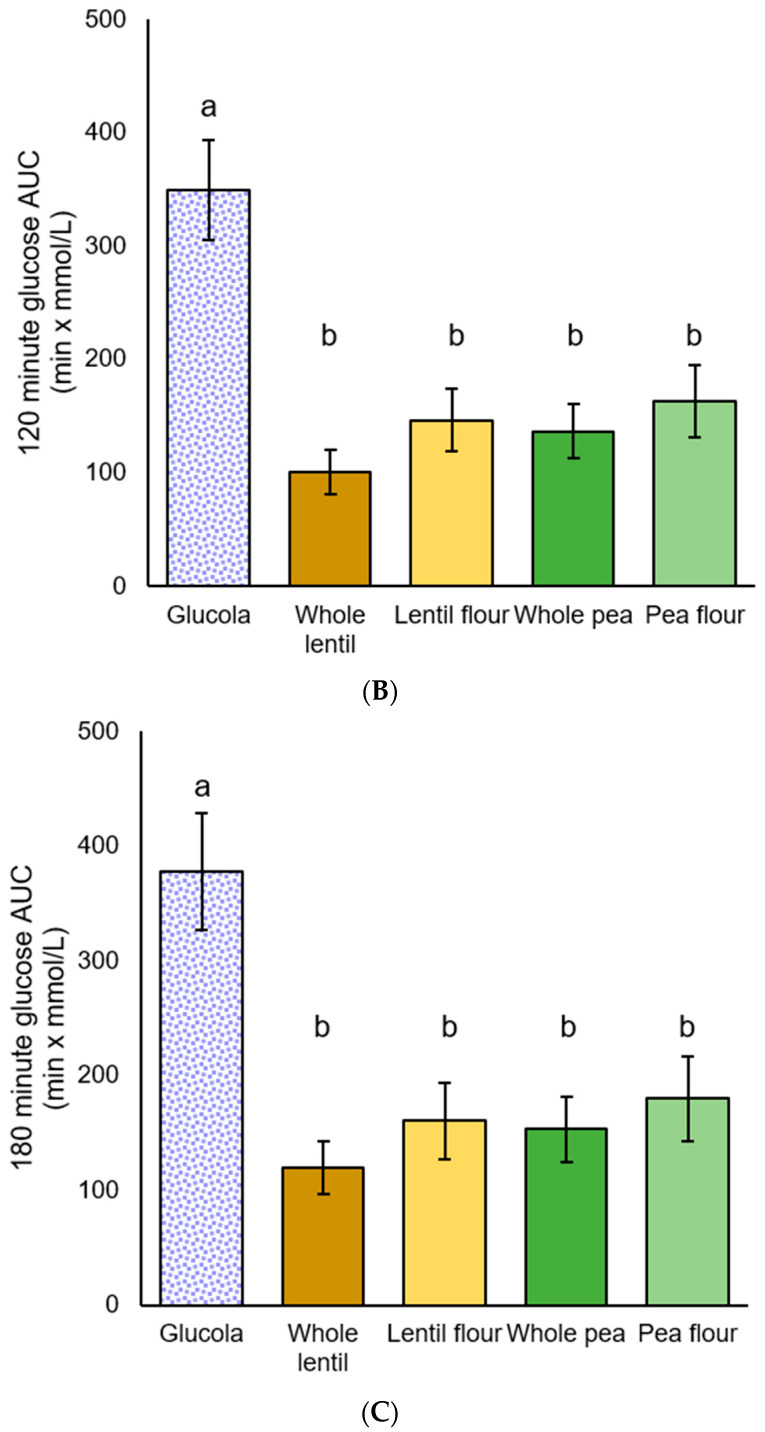
Plasma glucose iAUC for participants with metabolic syndrome: (**A**) 60 min, (**B**) 120 min, and (**C**) 180 min after the test meal consumption; data are presented as mean ± standard error of the mean. Letters that differ (a,b) indicate significant differences (*p* < 0.05) between treatments.

Among MetS participants, significant differences in iAUC glycemic response were observed by ANOVA [F(4,35) = 20.641, *p* < 0.001], [F(4,35) = 10.249, *p* < 0.001], [F(4,35) = 8.200, *p* < 0.001] at all of the timepoints. Post hoc iAUC main effect comparisons from 0 to 60 min were significantly greater for the Glucola treatment than for all four pulse treatments (all *p* < 0.001). Whole lentils exhibited a trend toward being lower than pea flour (*p* = 0.081) in the 0 to 60 min period. The 0 to 120 min glucose iAUC values showed a similar pattern, with *p* < 0.001 for three treatments, and *p* = 0.001 for pea flour. For the 0 to 180 min duration, glucose iAUC increment for Glucola was significantly different from whole lentil (*p* < 0.001), lentil flour and whole pea (*p* = 0.001), and pea flour (*p* = 0.004). There were no significant differences between the four test treatments across the 120 and 180 min iAUC time periods (Table 6, Figure 4).

### 3.3. Satiety

A statistically significant main effect was observed for hunger (*p* = 0.002), and a trend toward significance was noted with the overall appetite score (*p* = 0.06) among the T2DM participants. However, all post hoc analyses were not significant at *p* > 0.05 (Supplemental Table S3). With the MetS participants, statistically significant main effects were observed for hunger (*p* = 0.002), satiety (*p* < 0.001), volume one could eat (*p* = 0.010), and average appetite score (*p* = 0.001). Post hoc analysis indicated that overall appetite, satiety, hunger, desire to eat, and volume one could eat did not differ between treatments. The MetS cohort reported a reduced sense of fullness during testing after consuming the Glucola control as compared to whole lentil (*p* = 0.03), lentil flour (*p* = 0.03), whole pea (*p* = 0.01), and pea flour (*p* = 0.02) (Table 7). Means for the individual appetite components by study treatment, time, for the MetS cohort are shown in Supplemental Table S4.

### 3.4. Gastrointestinal Symptoms

About 20–30% of the T2DM and MetS participants experienced increased flatulence and/or bloating from eating the test meals. There were no significant differences between the study group types, or between the meal types and the control beverage. The pre-test meal gastrointestinal report was not included in the analysis. Table 7 shows the frequency distributions for symptoms by meal type for the pooled data from the T2DM and MetS groups.

## 4. Discussion

This 5 × 5 crossover study conducted among individuals with T2DM and MetS showed differences in postprandial glycemic response to whole pulses compared to pulse flour. The findings demonstrate that all four pulse treatments resulted in a lower glycemic response than the control glucose beverage from 0 to 120 min for T2DM and 0–180 min for MetS participants, aligned with the first hypothesis. However, at 60 min, a lower glycemic response in whole pulses was observed compared to the pea flour for participants with T2DM, while no differences between treatments were seen in those with MetS. Thus, the second hypothesis was demonstrated only in the T2DM group. Previous researchers have investigated the relationship between pulse flours and postprandial glycemic response, proposing mixed findings on whether these products help lower glucose response. This study adds to the research on glycemic response and pulse form by demonstrating higher glycemic response among individuals with T2DM or MetS with pulse flour.

Participant responses to dietary treatments in this study varied by disease state. While individuals with T2DM exhibited heightened glycemic responses to pulse flours, there was only a trend toward statistical significance in the elicited response among individuals with MetS. Previous research has shown that normoglycemic individuals may have an elevated postprandial glucose response to pulse flours [30,37]. Therefore, further examination of pulse flour effects on persons with MetS is warranted. Regardless, the current study results suggest pulse flours may disproportionately increase postprandial glycemia among those with T2DM. Some dietitians and nutritionists may advise pulse consumption to reduce the risk of T2DM, or for blood sugar management in those who have T2DM without regard to form of the pulse [61]. Pulses, when consumed alone or used in combination with higher glycemic foods such as rice, were shown to improve glycemic response in persons with T2DM [13]. However, this benefit may apply only to whole pulses, as pulse spreads did not reduce glycemic response when paired with higher glycemic foods [28]. Regular pulse consumption may also have long-term benefits for individuals with T2DM, with one recent meta-analysis showing improved longitudinal changes in blood glucose > 2.5 mmol/L. However, the efficacy of this improvement was reduced by half in studies that utilized pulse flours rather than their whole alternative [12].

One explanation for the elevated glycemic response to pulse flours may be found in the structural composition of the whole pulse. Cell wall integrity is broken during the flour milling process. An intact cell wall is resistant to digestion in the small intestine, thus slowing breakdown of carbohydrates and reducing the glucose response after consumption [11,24]. Milling pulses to create flour lowers these protective actions of the cell walls, increasing starch availability and resulting in glucose spikes after consumption [62]. The cell wall may be broken entirely during processing, in which case the starch granule is damaged, thus making it more available to enzymatic degradation. While the mechanisms involved in flour processing may help explain variances in glycemic response compared to whole pulses, there were also slight differences in the response by pulse type. The content of soluble and insoluble fibers varies between pulses. Therefore, each pulse has a unique impact on blood glucose. Ultimately, different pulses elicit variable glycemic responses [10,11]. This study used an equivalent amount of pulse flour to a ½ cup of whole pulse to control for pulse form while retaining nutrients. Smaller amounts of commercially processed pulse flour may cause less of a glycemic response, but would contain less of the health beneficial nutrients and fiber of the pulse flours used in this research.

The pulse treatments resulted in improved glycemic response compared to the control Glucola beverage, and it was also observed in the MetS group that the pulse treatments improved fullness. These treatments had beneficial impacts on postprandial glycemic response and fullness, and few gastrointestinal effects were reported. One randomized crossover trial showed that less than 50% of individuals noted an increase in flatulence after consuming pinto or baked beans, and only 19% noted an increase after consuming black-eyed-peas [40]. These findings are comparable to our current trial, in which only 20–30% of participants noted an increase in flatulence after any meal, with no significant differences observed compared to the control Glucola beverage. While the occurrence of flatulence after pulse consumption may be low, public perception of whether pulses as an ingredient cause gastrointestinal symptoms needs to be investigated in other studies.

### Limitations and Strengths

The study findings suggest awareness in consuming high amounts of pulse flour for persons with T2DM and possibly MetS. Although the sample size was theoretically adequate for a priori analysis for the T2DM group, the MetS sample was smaller than desired. The range of variation in age, body sizes, and medication dosages may have introduced variation in glycemic response, although the crossover study design mitigated individual differences. Due to the sample demographics of this study, these findings may not be generalizable to non-Whites or to younger adults with these conditions, or are not ambulatory, or have other comorbidities. Postprandial response to the specific flour and whole pulse products obtained from the Avondale lentil and Hampton pea were determined in this study and may not represent other lentil and pea varieties. Furthermore, commercial lentil or pea flours may not be processed in the same manner as described in this study. Therefore, other cultivars and processing methods may produce flour that may exhibit different effects on glycemic response. Insulin response, and other biomarkers of disease risk such as LDL, HDL, and triglycerides were not measured in this acute study. Future research should examine differences in these postprandial to determine if there are differences between whole pulses and their flour.

Strengths of the study include the equivalence of the whole pulse and pulse flour treatments, controlling for gene by environment nutrient interactions with seed grown in the same location, and using known genetic varieties. Few studies have compared pulse flour to whole pulses with or without additional processing. Nutrient analysis on the test foods was performed independently, not drawn from food labels. Another strength was the limited medication use in participants. Use of venous draws from an in-dwelling catheter reduced measurement variation and improved accuracy compared to finger-prick capillary blood sampling [63].

## 5. Conclusions

This study is one of the first to provide results on the effect of whole pulses and their equivalent flour counterparts on glycemic response in adults with T2DM and MetS. While pulse flours elicited a heightened glycemic response compared to the whole pulses, they may still be a viable option to lower postprandial blood glucose compared to cereal grain or other high glycemic food ingredients. Our findings support the concept that whole pulses or pulse flours may reduce postprandial glycemic response and should be incorporated into counseling recommendations for persons with metabolic diseases such as T2DM or MetS.

## Figures and Tables

**Table 1:** Percentage distribution of lentil and pea flour particle size.

Particle Size	Lentil Flour (%)	Pea Flour (%)
Above 177 µm	3.4	5.1
150–176 µm	28.5	61.6
149 µm and smaller	61.1	33.3

**Table 2:** Nutrient composition of T2DM and MetS test meals.

T2DM COHORT	Glucola	Whole Lentil	Lentil Flour	Whole Pea	PEA Flour
Total weight (g)	215	295.6	339.0	295.6	335.7
Pasta sauce (g)	--	166.6	166.6	166.6	166.6
Bread (g)	--	49.0	49.0	49.0	49.0
Pulses (g)	--	80.0	40.1	76.0	32.8
Water (g)	--	--	83.3	--	83.3
Sugar (g)	--	--	--	4.0	4.0
Energy (kcal)	200	391.0	392.0	382.0	383.0
Total carbohydrate (g)	50	66.8	66.0	68.2	67.7
Fiber (g)	0	11.6	13.9	12.3	11.5
Available CHO (g)	50	55.2	52.1	55.9	56.3
Pasta sauce (g)	--	15.6	15.6	15.6	15.6
Bread (g)	--	20.0	20.0	20.0	20.0
Pulse (g)	--	19.6	16.6	16.3	16.7
Sugar	--	--	--	4.0	4.0
Protein (g)	0	17.0	17.6	13.8	13.8
Fat (g)	0	6.1	6.3	5.9	6.3
METS COHORT					
Total weight (g)	215	291.6	335.0	287.6	327.7
Pasta Sauce (g)	--	166.6	166.6	166.6	166.6
Bread (g)	--	45.0	45.0	45.0	45.0
Pulse (g)	--	80.0	40.1	76.0	32.8
Water (g)	--	--	83.3	--	83.3
Energy (kcal)	200	380.0	380.9	355	356.4
Total carbohydrate (g)	50	64.9	64.2	62.3	61.9
Fiber (g)	0	11.3	13.6	12.1	11.2
Available CHO (g)	50	53.6	50.5	50.3	50.6
Pasta Sauce (g)		15.6	15.6	15.6	15.6
Bread (g)		18.3	18.3	18.3	18.3
Pulse (g)	-	19.6	16.6	16.3	16.7
Protein (g)	0	16.6	17.1	13.4	13.4
Fat (g)	0	5.9	6.1	5.7	6.1

CHO = carbohydrate, g = grams, kcal = kilocalorie.

**Table 3:** Descriptive characteristics of study participants (*n* = 19).

Variable	All	T2DM	MetS	*p*
	$\bar{X}$ ± SEM			
Age (yrs)	51 ± 3	55 ± 4	44 ± 3	0.07
Weight (kg) ^1^	101.6 ± 3.8	101.1 ± 5.5	102.3 ± 5.2	0.88
Height (cm) ^1^	172.2 ± 2.1	173.4 ± 3.0	170.7 ± 3.1	0.55
BMI (kg/m^2^) ^1^	34.1 ± 0.7	33.4 ± 1.0	35.0 ± 1.1	0.31
HbA1c (%)	6.2 ± 0.2	6.5 ± 0.2	5.9 ± 0.1	0.07
Glucose (mmol/L)	6.7 ± 0.4	7.3 ± 0.6	5.9 ± 0.2	0.05
Waist circumference (cm)	114.7 ± 2.7	115.5 ± 4.3	113.7 ± 3.0	0.76
Total Cholesterol (mmol/L)	4.7 ± 0.3	4.1 ± 0.3	5.5 ± 0.4	0.01
LDL-C (mmol/L)	2.7 ± 0.3	2.2 ± 0.3	3.4 ± 0.4	0.01
HDL-C (mmol/L)	1.1 ± 0.1	1.1 ± 0.1	1.1 ± 0.1	0.56
Triglycerides (mmol/L)	2.3 ± 0.2	2.3 ± 0.4	2.3 ± 0.4	0.98
Systolic blood pressure (mm Hg)	126.8 ± 2.7	129.6 ± 3.6	123.0 ± 4.0	0.24
Diastolic blood pressure (mm Hg)	82.8 ± 1.4	81.4 ± 1.2	84.8 ± 2.9	0.25

^1^ Values obtained at study entry. Differences between T2DM and MetS participants were evaluated with independent *t*-test. BMI = body mass index, HbA1c = hemoglobin A1c, LDL-C = low density lipoprotein cholesterol, HDL-C = high density lipoprotein cholesterol.

**Table 4:** Incremental changes in plasma glucose concentrations among participants ^1^.

	Glucola	Whole Lentil	Lentil Flour	Whole Pea	Pea Flour
T2DM					
Fasting	7.2 ± 0.6	7.3 ± 0.6	7.4 ± 0.4	7.2 ± 0.5	7.1 ± 0.5
30 min	11.3 ± 0.7	8.4 ± 0.6	9.7 ± 0.6	8.9 ± 0.6	10.2 ± 0.7
60 min	13.4 ± 0.8	9.8 ± 0.8	11.2 ± 0.8	10.1 ± 0.8	11.0 ± 0.9
90 min	12.1 ± 0.9	9.4 ± 0.8	10.9 ± 0.8	10.0 ± 0.8	10.1 ± 0.9
120 min	9.7 ± 0.9	8.3 ± 0.8	9.7 ± 0.8	8.8 ± 0.7	8.4 ± 0.9
150 min	7.8 ± 0.8	7.3 ± 0.7	8.5 ± 0.8	7.5 ± 0.7	7.5 ± 0.9
180 min	6.8 ± 0.7	6.6 ± 0.7	7.4 ± 0.7	6.8 ± 0.7	6.6 ± 0.7
METS					
Fasting	5.8 ± 0.2	5.6 ± 0.2	6.1 ± 0.2	5.6 ± 0.2	5.8 ± 0.2
30 min	9.2 ± 0.5	6.2 ± 0.3	7.8 ± 0.3	6.4 ± 0.3	7.6 ± 0.3
60 min	10.5 ± 0.8	7.0 ± 0.3	7.9 ± 0.4	7.5 ± 0.4	7.8 ± 0.6
90 min	8.6 ± 0.7	6.6 ± 0.4	7.2 ± 0.4	7.0 ± 0.4	6.9 ± 0.6
120 min	6.6 ± 0.5	6.1 ± 0.3	6.5 ± 0.3	6.3 ± 0.2	6.1 ± 0.2
150 min	5.3 ± 0.5	5.6 ± 0.3	6.0 ± 0.2	5.5 ± 0.2	5.3 ± 0.3
180 min	4.5 ± 0.3	5.2 ± 0.3	5.1 ± 0.2	5.1 ± 0.2	4.8 ± 0.2

^1^ All values are means ± standard error of the mean (mmol/L). T2DM = Type 2 diabetes mellitus. METS = Metabolic syndrome. Min = minutes.

**Table 5:** Postprandial incremental areas under the curve for plasma glucose among participants with type 2 diabetes (*n* = 11).

	Glucola	Whole Lentil	Lentil Flour	Whole Pea	Pea Flour
60 min iAUC	217.5 ± 17.7	73.4 ± 10.6	124.6 ± 16.9	91.5 ± 10.5	150.9 ± 14.9
120 min iAUC	494.2 ± 40.6	192.9 ± 30.5	316.7 ± 47.6	241.7 ± 30.2	319.7 ± 39.8
180 min iAUC	564.8 ± 56.7	223.5 ± 38.2	394.6 ± 71.1	280.1 ± 41.9	369.7 ± 53.5

Data are presented as mean ± standard error of the mean, mol × min/L (calculated by the trapezoidal rule).

**Table 6:** Postprandial areas under the curve for plasma glucose among participants with metabolic syndrome ^1^.

	Glucola	Whole Lentil	Lentil Flour	Whole Pea	Pea Flour
60 min iAUC	173.4 ± 18.3	39.8 ± 5.6	77.4 ± 9.1	54.0 ± 8.7	82.9 ± 11.6
120 min iAUC	349.7 ± 43.9	101.1 ± 19.6	146.3 ± 27.5	136.5 ± 24.1	162.9 ± 32.1
180 min iAUC	377.4 ± 50.8	119.4 ± 23.4	160.2 ± 33.0	153.1 ± 28.6	179.7 ± 37.4

^1^ Data are presented as mean ± standard error of the mean, mol × min/L (calculated by the trapezoidal rule).

**Table 7:** Frequency reports of gastrointestinal symptoms for both T2DM and MetS cohorts.

	Meal Average	Glucola	Whole Lentils	Lentil Flour	Whole Peas	Pea Flour
			**% (*n*)**			
Flatulence						
No Change	73.9 (65)	88.9 (16)	70.6 (12)	68.8 (11)	66.7 (12)	73.7 (14)
Increased	26.1 (23)	11.1 (2)	29.4 (5)	31.3 (5)	33.3 (6)	26.3 (5)
Decreased	0	0	0	0	0	0
Bloating						
No Change	80.7 (71)	83.3 (15)	88.2 (15)	81.3 (13)	77.8 (14)	73.7 (14)
Increased	19.3 (17)	16.7 (3)	11.8 (2)	18.8 (3)	22.2 (4)	26.3 (5)
Decreased	0	0	0	0	0	0

Data are the percentage and number of cases indicating yes for a given question.

## Data Availability

The data presented in this study are available on request from the corresponding author. The data are not publicly available due to privacy restrictions.

## Supplementary Materials

The following supporting information can be downloaded at https://www.mdpi.com/article/10.3390/foods14111933/s1, Table S1: Methodology and calculations for dry weight basis equivalency of lentils and peas; Table S2: Nutrient composition of pre-test evening meals; Table S3: Satiety and overall appetite scores for participants with type 2 diabetes mellitus (T2DM); Table S4: Satiety and overall appetite scores for participants with metabolic syndrome (MetS).
