# Supplementary material for: Pea and Lentil Flours Increase Postprandial Glycemic Response in Adults with Type 2 Diabetes and Metabolic Syndrome
Source: Foods. 2025 May 29;14(11):1933. doi: 10.3390/foods14111933 (PMC12154248; doi:10.3390/foods14111933)
Supplement: Supplementary file 1 [file foods-14-01933-s001.zip › foods-3659131-supplementary.pdf]

Supplemental Table S1. Methodology and calculations for dry weight basis equivalency of lentils and peas

Lentils:

One cup of cooked lentils were determined to be 160 g by measuring three different samples after cooking. For the ½ cup serving, 80 g was weighed out for the whole lentil treatment.

To determine an equivalent ½ cup dry weight basis amount for lentil flour, the following calculations were made based on moisture content values from Eurofins analysis. The moisture content for the samples were 54.9% for the boiled lentils and 10.1% for the lentil flour:

Amount of dry solids in 100 g cooked lentils = 100 g – 54.9 g = 45.1 g dry solids per 100 g cooked lentils
Amount of dry solids in 160 g cooked lentils per cup = 160 g cooked lentils/cup * 45.1 g dry solids/100 g cooked = 72.16 dry cooked lentil solids per cup
Amount of dry solids in 100 g lentil flour = 100 g – 10.1 g = 89.9 g dry solids per 100 g lentil flour
Flour equivalent amount to cooked lentils = 72.16 g dry cooked lentils solids per 1 cup/(89.9 g dry solids/100 g lentil flour) = 80.27 g dry lentil flour solids per 1 cup

Thus, 40.13 g of lentil flour was used for an equivalent ½ cup dry weight basis to cooked lentils.

Peas:

One cup of cooked peas were determined to be 152 g, meaning that ½ cup was 76 g. This is the pulse amount used for the whole pea treatment.

To determine an equivalent ½ cup dry weight basis amount for pea flour, the following calculations were made based on moisture content values from Eurofins analysis. The moisture content for the samples were 60.8% for the boiled lentils and 9.2% for the pea flour:
Amount of dry solids in 100 g cooked peas = 100 g – 60.8 g = 39.2 g dry solids per 100 g cooked peas
Amount of dry solids in 152 g cooked peas per cup = 152 g cooked peas/cup * 39.2 g dry solids/100 g cooked = 59.58 g dry cooked pea solids per cup
Amount of dry solids in 100 g pea flour = 100 g – 9.2 g = 90.8 g dry solids per 100 g pea flour
Flour equivalent amount to cooked peas = 59.58 g dry cooked pea solids per 1 cup /(90.8 g dry solids/100 g pea flour) = 65.62 g dry pea flour solids per 1 cup

Thus, 32.81 g of pea flour was used for an equivalent ½ cup dry weight serving to cooked peas.

Supplemental Table S2. Nutrient composition of pre-test evening meals.

	Honey Roasted Turkey	Salisbury Steak	Roasted Turkey and Stuffing	Nabisco® Teddy Grahams	Pepperidge Farm® Milano
Total Calories (g)	280	480	290	120	110
Fat (g)	6	22	7	4	6
CHO (g)	33	48	37	21	14
Dietary Fiber (g)	5	6	5	1	1
Net CHO (g)	28	42	32	20	13
Protein (g)	21	23	20	2	1
Sodium (mg)	860	1190	1110	90	40

CHO = carbohydrate, g = grams

Supplemental Table S3. Satiety and overall appetite scores for participants with Type 2 diabetes mellitus (T2DM).[1]

		Baseline	60 minutes	120 minutes	180 minutes
Hunger					
	Glucola	48.7 ± 7.8	56.2 ± 5.6	70.0 ± 5.1	73.7 ± 7.4
	Whole Lentil	75.8 ± 6.9	28.0 ± 10.4	44.0 ± 14.0	52.0 ± 15.1
	Lentil Flour	61.6 ± 7.7	28.1 ± 4.6	48.3 ± 7.6	69.3 ± 4.4
	Whole Pea	52.1 ± 10.9	26.9 ± 7.2	38.0 ± 8.5	53.8 ± 9.2
	Pea Flour	53.0 ± 10.7	21.1 ± 7.3	41.1 ± 5.7	61.9 ± 8.5
Fullness					
	Glucola	31.3 ± 6.6	34.9 ± 6.3	22.1 ± 8.1	18.5 ± 5.2
	Whole Lentil	29.6 ± 9.0	68.6 ± 8.8	55.1 ± 14.5	48.8 ± 15.3
	Lentil Flour	33.9 ± 7.0	68.7 ± 3.7	57.1 ± 6.0	32.9 ± 7.5
	Whole Pea	41.3 ± 8.1	70.3 ± 4.9	59.8 ± 7.1	44.1 ± 8.9
	Pea Flour	34.4 ± 10.9	67.9 ± 9.1	55.7 ± 5.6	37.1 ± 8.4
Satiety					
	Glucola	31.4 ± 5.5	29.8 ± 4.6	22.4 ± 7.6	24.6 ± 6.0
	Whole Lentil	31.5 ± 8.8	62.0 ± 14.2	55.5 ± 15.8	48.0 ± 17.4
	Lentil Flour	33.7 ± 5.3	55.8 ± 6.8	44.1 ± 5.3	35.3 ± 5.0
	Whole Pea	38.6 ± 6.2	60.1 ± 8.0	53.1 ± 8.3	47.75 ± 7.4
	Pea Flour	39.4 ± 12.0	71.2 ± 6.9	53.0 ± 6.4	45.9 ± 10.1
Desire to Eat					
	Glucola	53.3 ± 10.9	64.3 ± 5.8	73.9 ± 6.9	77.2 ± 8.2
	Whole Lentil	73.7 ± 8.4	30.4 ± 13.6	42.7 ± 15.8	54.6 ± 15.9
	Lentil Flour	63.3 ± 8.7	30.6 ± 5.8	49.1 ± 8.0	72.8 ± 5.4
	Whole Pea	54.4 ± 8.2	26.1 ± 8.0	35.0 ± 8.8	57.4 ± 9.5
	Pea Flour	52.0 ± 12.5	27.1 ± 9.4	49.5 ± 6.7	61.1 ± 9.6
Volume one could eat					
	Glucola	63.6 ± 6.8	64.2 ± 6.6	70.4 ± 7.0	75.9 ± 6.2
	Whole Lentil	68.8 ± 7.6	30.6 ± 9.7	53.8 ± 15.8	57.4 ± 16.6
	Lentil Flour	63.1 ± 7.6	35.3 ± 5.9	52.6 ± 7.7	71.2 ± 5.5
	Whole Pea	57.3 ± 7.5	29.8 ± 6.7	42.1 ± 8.1	55.4 ± 9.4
	Pea Flour	57.3 ± 9.2	42.0 ± 10.1	48.6 ± 3.7	68.0 ± 5.5
Average appetite score					
	Glucola	58.6 ± 5.6	62.5 ± 5.6	73.1 ± 6.7	77.1 ± 6.6
	Whole Lentil	72.2 ± 6.9	30.1 ± 9.9	46.4 ± 14.3	53.8 ± 15.4
	Lentil Flour	63.5 ± 6.3	31.3 ± 4.3	48.2 ± 6.7	70.1 ± 4.5
	Whole Pea	55.6 ± 8.5	28.1 ± 6.3	38.8 ± 7.9	55.6 ± 9.1
	Pea Flour	57.0 ± 10.4	30.6 ± 8.3	45.9 ± 4.4	63.5 ± 7.7

[1]All values are means ± standard error of the mean.

Supplemental Table S4. Satiety and overall appetite scores for participants with Metabolic Syndrome (MetS).[1]

		Baseline	60 minutes	120 minutes	180 minutes
Hunger					
	Glucola	35.5 ± 9.2	44.6 ± 6.5	52.8 ± 6.9	68.9 ± 5.2
	Whole Lentil	64.9 ± 7.5	28.7 ± 3.1	43.4 ± 5.9	47.8 ± 6.6
	Lentil Flour	57.3 ± 8.5	29.6 ± 8.6	49.8 ± 5.7	56.8 ± 5.1
	Whole Pea	49.4 ± 7.9	32.8 ± 6.6	32.5 ± 5.0	56.5 ± 4.3
	Pea Flour	50.3 ± 9.1	17.3 ± 4.5	29.5 ± 4.0	44.4 ± 5.6
Fullness					
	Glucola[2]	34.9 ± 4.9	39.5 ± 8.2	35.0 ± 7.7	22.7 ± 7.5
	Whole Lentil	24.9 ± 6.3	69.3 ± 3.4	62.0 ± 4.8	50.4 ± 4.8
	Lentil Flour	30.4 ± 8.2	73.4 ± 2.8	58.4 ± 5.5	46.4 ± 5.9
	Whole Pea	56.0 ± 23.3	58.6 ± 5.2	53.3 ± 4.9	47.5 ± 4.8
	Pea Flour	29.3 ± 5.9	67.9 ± 5.3	59.3 ± 4.6	55.1 ± 4.8
Satiety					
	Glucola	48.3 ± 5.7	44.3 ± 4.5	41.1 ± 6.5	25.1 ± 6.7
	Whole Lentil	28.1 ± 5.8	66.4 ± 4.3	55.6 ± 4.6	48.5 ± 4.4
	Lentil Flour	30.5 ± 5.1	70.9 ± 5.4	53.8 ± 6.3	45.0 ± 5.2
	Whole Pea	36.9 ± 3.9	55.9 ± 4.9	47.4 ± 5.0	44.2 ± 6.4
	Pea Flour	31.8 ± 6.0	65.2 ± 6.2	61.1 ± 4.1	51.1 ± 8.0
Desire to Eat					
	Glucola	45.4 ± 9.5	43.6 ± 7.6	55.6 ± 5.7	75.8 ± 4.7
	Whole Lentil	63.9 ± 7.2	31.9 ± 7.7	43.9 ± 6.5	55.8 ± 8.1
	Lentil Flour	60.8 ± 9.0	26.1 ± 7.4	49.3 ± 7.5	59.8 ± 6.7
	Whole Pea	81.4 ± 19.7	34.95 ± 7.7	41.9 ± 5.8	57.9 ± 5.1
	Pea Flour	54.6 ± 8.9	23.1 ± 8.5	38.5 ± 5.8	51.6 ± 6.7
Volume one could eat					
	Glucola	50.5 ± 7.6	51.1 ± 3.6	53.7 ± 3.4	66.4 ± 4.7
	Whole Lentil	61.4 ± 6.2	35.3 ± 4.6	46.5 ± 5.0	54.1 ± 6.1
	Lentil Flour	61.9 ± 6.1	26.7 ± 6.9	49.1 ± 5.3	60.1 ± 5.4
	Whole Pea	57.6 ± 3.4	41.4 ± 2.9	53.8 ± 4.0	58.5 ± 4.9
	Pea Flour	57.0 ± 4.8	29.3 ± 6.3	39.1 ± 6.6	49.9 ± 8.3
Average appetite score					
	Glucola	49.1 ± 7.0	50.0 ± 3.4	56.8 ± 3.5	72.1 ± 4.4
	Whole Lentil	66.3 ± 5.6	31.6 ± 3.9	43.0 ± 4.3	51.8 ± 6.2
	Lentil Flour	62.4 ± 7.6	27.2 ± 6.0	47.4 ± 5.7	57.5 ± 5.2
	Whole Pea	58.1 ± 4.6	37.6 ± 5.0	43.7 ± 3.4	56.3 ± 3.8
	Pea Flour	58.2 ± 5.3	25.4 ± 5.4	37.0 ± 3.9	47.7 ± 5.6

[1]All values are means ± standard error of the mean. [2]Significantly less than whole lentil ($p = 0.03$), lentil flour ($p = 0.03$), whole pea ($p = 0.01$), pea flour ($p = 0.02$).
